# Systemic Use of Oral Rapamycin, Prednisone and Colchicine After Coronary Bare-Metal Stent Implantation: Narrative Review of Randomized Clinical Trials

**DOI:** 10.3390/biomedicines14081685

**Published:** 2026-07-27

**Authors:** Carlos Fernandez-Pereira, Alfredo E. Rodriguez

**Affiliations:** Cardiac Unit, Cardiovascular Research Center (CECI), Otamendi Hospital, Buenos Aires 1001, Argentina; cfernandezpereira@centroceci.com.ar

**Keywords:** bare-metal stents, rapamycin, sirolimus, colchicine, anti-inflammatory therapy, cost-effective cardiology, percutaneous coronary intervention

## Abstract

**Background:** Coronary stenting remains the cornerstone of interventional cardiology. Drug-eluting stents (DES) have reduced restenosis compared to bare-metal stents (BMS), but their high cost and need for prolonged dual antiplatelet therapy (DAPT) limit accessibility in many regions. Recent evidence has revisited the potential role of systemic pharmacologic adjuncts—oral sirolimus (rapamycin), prednisone and colchicine—as cost-effective therapies to mitigate restenosis and adverse events following BMS implantation. **Objective:** This review critically evaluates the clinical and mechanistic evidence supporting the use of oral immunosuppressive or anti-inflammatory drugs following BMS implantation, with emphasis on their applicability in both resource-limited and general cardiology settings. Three randomized clinical trials comparing this strategy against DES are analyzed and discussed in this review. **Conclusions:** Oral sirolimus, prednisone, and colchicine demonstrate promising anti-inflammatory and antiproliferative effects that translate into clinical outcomes comparable to DES in a highly select population. Their systemic administration following BMS implantation may provide a feasible, cost-effective alternative where DES use is restricted by cost or clinical contraindications. Larger-scale, long-term trials are warranted to confirm safety, optimize dosing, and identify ideal patient populations for this “pharmacologic stent hybrid” strategy.

## 1. Introduction

Percutaneous coronary intervention (PCI) with stent implantation has changed the management of obstructive coronary artery disease (CAD). Initially, bare-metal stents (BMS) significantly reduced acute vessel recoil and restenosis compared with balloon angioplasty alone [1]. Nevertheless, in-stent restenosis (ISR) due to intimal hyperplasia remained a major limitation, affecting 20–30% of patients in early series [2]. The subsequent introduction of first-generation drug-eluting stents (DES), releasing sirolimus or paclitaxel locally, markedly decreased restenosis rates but introduced new challenges, such as late stent thrombosis, prolonged requirement for dual antiplatelet therapy (DAPT), and elevated costs [3,4].

Despite new DES designs being significantly safer than older ones, with DES late thrombosis rates less than 1% at 5 years in most randomized series [5,6,7,8], limitations, such as early neo-atherosclerosis and endothelial dysfunction, remain a problem with the second generation (DES2G). Both limitations could be linked to cardiac and non-cardiac adverse events observed in the late outcome after DES2G implantation [9,10,11,12,13,14,15].

While DES have become the standard of care in most developed settings, their widespread adoption remains limited in low- and middle-income countries (LMICs) due to affordability and access constraints [16]. Furthermore, certain clinical scenarios—such as high bleeding risk, need for early surgery, lesions in the saphenous vein graft, or limited life expectancy—may warrant reconsideration of BMS implantation if associated restenosis risk can be effectively managed. BMS accounts for about 22% of total coronary stent implantations globally, with annual usage above 4.5 million units [17].

The United States accounts for approximately 29% of global BMS utilization, sustained by more than 900,000 PCI procedures annually. BMS are used in 18% of U.S. coronary angioplasties, primarily in patients with high bleeding risk. Large-diameter arteries account for 64% of domestic usage. Hospital cath labs are performing 72% of procedures using BMS. Reduced DAPT time influences 69% of U.S. physicians’ preference, reinforcing the 2026 BMS Market Share.

Europe accounts for 26% of global demand, with guideline-driven use accounting for 63% of procedures. The region benefits from robust regulatory surveillance and adherence to clinical protocols, ensuring standardized stent utilization across healthcare systems. BMS are primarily used in cases requiring cost-efficiency or a particular patient profile. Public healthcare systems play a significant role in procurement decisions, emphasizing value-based care.

**Figure 1:**
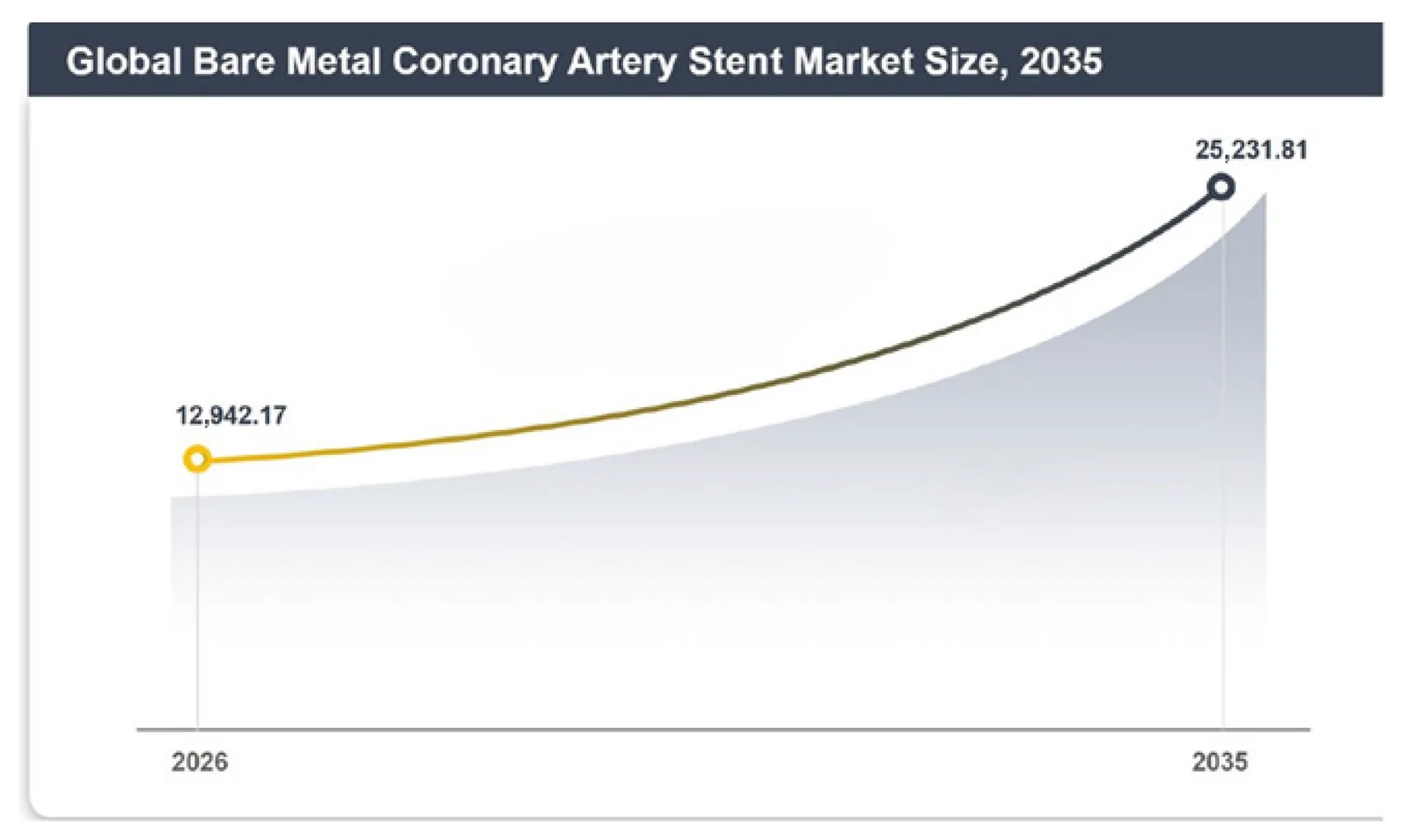
Forecast of Coronary Bare Metal Stent Market 2026/2035. Reference [17].

The expected Compound Annual Growth Rate for the coronary BMS market worldwide over the next 9 years, 2026 to 2035, is 7.7%, from USD 12,942.17 million to 25,231.81 million, as shown in Figure 1 [17].

The concept of systemic stent optimization—using oral pharmacologic agents to mimic the local antiproliferative effects of DES—has re-emerged in this context. Two pharmacologic agents, Sirolimus (also known as rapamycin) and Colchicine, have shown particular promise. Both possess potent anti-inflammatory and antiproliferative actions but differ mechanistically: sirolimus by inhibiting the mammalian target of the rapamycin (mTOR) pathway, and colchicine by suppressing microtubule-dependent inflammatory signaling [18,19,20,21].

This narrative review synthesizes all available data from randomized clinical trials (RCTs) on the use of oral immunosuppressive or anti-inflammatory therapy (sirolimus, prednisone, and colchicine) following BMS implantation, compared with DES, published in PubMed/Embase.

In the search, we used the keywords: “stents, randomized, DES,” and subsequently we added, one at a time, “oral rapamycin, prednisone, and colchicine”.

We found seventeen manuscripts: four were unrelated and thirteen related.

Of these thirteen, three were review articles, three were meta-analyses, and the other seven included the three RCTs presented here; we considered only the data from the longest available follow-up for each.

No other RCT comparing oral immunosuppressive or anti-inflammatory drugs plus BMS against DES was found.

## 2. Mechanistic Rationale for the Use of Systemic Therapy After BMS Implantation

### 2.1. The Biological Basis of Restenosis

Restenosis after BMS implantation results from a complex interplay of vascular injury, platelet activation, inflammation, and smooth muscle cell proliferation leading to neointimal hyperplasia. Endothelial denudation during stent deployment triggers the release of platelet-derived growth factor (PDGF), transforming growth factor β, and inflammatory cytokines, activating the mTOR signaling pathway that drives vascular smooth muscle migration and proliferation. In parallel, infiltration of neutrophils and macrophages promotes a pro-inflammatory environment via IL-1β and NLRP3 inflammasome activation [20].

### 2.2. Sirolimus Mechanism of Action, Pharmacokinetics and Dosing Considerations

Sirolimus is a macrolide originally developed as an antifungal agent, later found to strongly inhibit vascular smooth muscle proliferation by binding to the FK-binding protein.

FK stands for FK506 (Tacrolimus), which suppresses the immune system by binding to a protein called FK506-binding protein 12 (FKBP12). This drug–FKBP12 complex then binds directly to the FKBP-rapamycin-binding domain of the mechanistic target of rapamycin (mTOR). This halts mTOR Complex 1 (mTORC1) activity, stopping cell division and migration.

This mechanism suppresses cell cycle progression from G1 to S phase, reduces protein synthesis, and inhibits neointimal hyperplasia [18,19,20,21]. When administered systemically, sirolimus achieves sufficient plasma concentrations to exert these vascular effects without stent coating; this is the principle behind early oral sirolimus–BMS trials. Optimal dosing remains uncertain. Oral sirolimus exhibits variable bioavailability due to extensive hepatic metabolism (CYP3A4) and P-glycoprotein efflux. Early regimens used 2 mg daily for 14–30 days, targeting trough levels of 4–10 ng/mL, similar to kidney transplant protocols but of shorter duration [22]. Side effects were generally mild—aphthous ulcers, transient leukopenia, and hyperlipidemia—but required monitoring, especially in elderly patients or those on clopidogrel [23].

### 2.3. Oral Rapamycin Following BMS Implantation

The first preclinical data, published in 1999, evaluating the use of oral sirolimus after PCI with BMS implantation for the reduction in neointimal hyperplasia in animal restenosis models showed positive results [22]. This led to studies evaluating safety and feasibility in pilot trials [23,24,25,26] where patients with de novo coronary lesions received sirolimus for short periods immediately after BMS. These trials used a range of doses from 2 to 5 mg per day for 14–30 days, and both assessed the incidence of TLR and late loss by angiography at 6 months. The results demonstrated a significant reduction in binary restenosis, and the ORAR pilot studies are the first to establish a significant reduction in both parameters versus BMS alone, when sirolimus serum levels were >8 ng/mL [23,25]. Subsequently, other studies, including randomized RCTs in de novo coronary and ISR lesions, confirmed these favorable results [26,27].

In 2004, the OSIRIS randomized trial [27] evaluated this approach for the treatment of ISR, comparing a placebo versus different loading doses: one with a loading dose of 8 mg and the other with a high loading dose of 24 mg of sirolimus, both followed by 2 mg per day for a week. Consistent with other studies, target vessel revascularization (TVR) and restenosis were significantly reduced, and there was a significant correlation between the drug concentration in the blood on the day of the procedure and the reduction in late lumen loss at follow-up (*p* < 0.001).

The Oral Rapamacyn in ARgentina (ORAR) III trial was the 1st randomized comparison between BMS implantation followed by 14 days of oral sirolimus (OR) versus a DES strategy alone without adjunctive OR. In the DES group, they used 98% of 1st-DES designs; either Taxus (Boston Scientific Corporation, Natick, MA, USA), Endeavour (Medtronic Vascular, Santa Rosa, CA, USA) or Cypher, (Cordis, Warren, NJ, USA). Patients were followed at 1, 3 and 5 years after PCI [28,29,30,31,32,33]. The primary objectives were costs and TVR. Safety was defined as the composite of death, myocardial infarction (MI) and stroke. In the OR arm, patients received a sirolimus loading dose of 10 mg before the PCI procedure, followed by 3 mg daily for 13 days. OR patients received DAPT, clopidogrel 75 mg a day, and aspirin for 1 month, and DES patients for at least 1 year. At 1 and 3 years, both groups, DES and OR, had similar safety and efficacy, although the OR strategy was cost-saving [30,31,32]. At 5 years [32], unexpected major differences in clinical outcome between the two groups were observed. The incidence of all causes of death, and the composite of death, MI, and stroke was significant in favor of the group of OR plus BMS (*p* = 0.02 and 0.01, respectively) Table 1.

These differences were mostly driven by poor outcomes with DES in the elderly cohort of patients, a greater incidence of stent thrombosis, and cardiac death with DES. The presence of new malignancies was numerically higher in the DES group, 10% vs. 4% (*p* = 0.09). The cumulative cost remained higher in the DES group.

The gradual deterioration in results with these 1st-generation DES beyond 3 years has been observed in other experiences and is consistent with the ORAR III findings at 5 years [11,12,34].

However, the major limitation of these results observed in ORAR III was the use of 1st-generation DES, which were subsequently replaced by newer DES platforms that completely addressed the problem of stent thrombosis.

A systematic review evaluating the effectiveness of oral Sirolimus after BMS implantation to prevent restenosis was published more than 10 years ago. Ref. [35] included four RCTs in patients with de novo coronary lesions. Three studies were versus BMS alone, and the fourth was versus 1st-generation DES. Follow-up duration ranged from 7 months to 5 years, and the study showed that, compared with BMS alone, early short-term systemic use of Sirolimus after BMS led to a major reduction in target lesion revascularization (TLR) and adverse cardiac events.

Although limited by small sample sizes, these trials collectively suggest that oral Sirolimus can provide outcomes comparable to DES at a fraction of the cost, rendering it a viable option in settings where DES is unavailable or unaffordable. However, the lack of large-scale, multicenter randomized trials, driven mainly by industry interests and logistical challenges in ensuring adherence and monitoring blood levels, has constrained broader adoption.

### 2.4. Corticosteroids

Preclinical data showed a reduction in neointimal hyperplasia after 2 weeks of continuous hydrocortisone infusion following aortic balloon injury, whereas initial clinical studies did not yield similar results. Possible reasons for failure were that they were performed in the pre-stent era and the steroids were given for a short period of time [36,37].

However, after BMS implantation, small trials showed that prednisone was effective, reducing angiographic and clinical restenosis. The first one [38,39] evaluated a selected group of non-diabetic patients with CAD and an elevated C-reactive protein (CRP) 72 h after successful PCI with a BMS, who were randomized to either high and decreased doses of oral prednisone or placebo for 45 days. The 12-month event rate was more frequently seen with placebo (*p* = 0.006), driven by a higher rate of ISR-related repeat procedures.

The same investigators went on to present an RCT, the CEREA-DES trial [40], in which participants were randomized to three arms: BMS plus placebo (n = 125), BMS plus prednisone (n = 122), and DES alone (n = 127). Prednisone was given orally for 40 days. At 1 year, the authors found that patients receiving BMS alone had lower free clinical adverse event survival than those treated with prednisone or DES: 80.8% in the controls, compared with 88.0% with prednisone and 88.8% in the DES group (*p* = 0.04 and 0.006, respectively). At 4 years of follow-up, patients receiving a BMS alone had significantly poorer event-free survival compared with prednisone (*p* = 0.007) and/or DES (*p* = 0.03).

DES patients suffered more very late stent thrombosis and MI. The need for TVR remained similar in the prednisone and DES groups. Therefore, in this non-diabetic population, prednisone therapy compared to BMS alone improved event-free survival at 1 and 4 years and was similar compared to DES. Although around 15% of patients suffered minor drug-related side effects, none of them discontinued the treatment (Table 2).

However, as with all steroids, a limitation on the use of prednisone was the exclusion of diabetic patients, which is the highest-risk group for restenosis after stent implantation. As in previous experience with oral sirolimus, the use of 1st DES designs, Cypher and Taxus as comparators was a major limitation of this trial.

#### Meta-Analysis

A patient-level meta-analysis was published [41] using data from all eligible RCTs, using OI therapy with either sirolimus or prednisone for the prevention of restenosis at that time. A total of 1246 patients (608 randomized to BMS plus OI and 638 randomized to BMS/DES and no OI) and 1456 lesions (711 randomized to BMS plus OI and 745 randomized to BMS/DES) were included. The meta-analysis found a decrease in the risk of TLR/TVR compared with the control arms, and oral therapy also reduced late luminal loss in patients with follow-up angiograms, providing a biologic explanation for these results.

The authors of this meta-analysis concluded that, in patients undergoing PCI, adding oral immunosuppressive therapy to BMS reduced the risk of TVR compared with BMS alone, and this effect was similar to that of DES.

### 2.5. Colchicine

#### Mechanism of Action

Colchicine, a well-established anti-inflammatory compound used in gout and pericarditis, disrupts microtubule assembly, thereby preventing neutrophil chemotaxis, inflammasome activation, and IL-1β release. Colchicine also downregulates vascular cell adhesion molecule-1 (VCAM-1) and reduces endothelial dysfunction, potentially mitigating post-stent vascular inflammation [18,42,43,44]. These properties have been linked to significant reductions in recurrent ischemic events in clinical trials such as COLCOT and LoDoCo2 [45,46].

It has been evaluated for ISR prevention due to its ability to inhibit neointimal hyperplasia. As with other oral agents, initial studies in the pre-stent era failed to demonstrate efficacy [47].

### 2.6. Colchicine Following Stent Implantation

Following the introduction of BMS, it was anticipated that it might reduce restenosis given its effects on limiting intimal hyperplasia. Deftereos et al., in a double-blind, prospective, placebo-controlled RCT [48], assessed colchicine’s effect preventing ISR in a high-risk population of diabetic patients with CAD and contraindications for DES. ISR was measured by angiography and with intravascular ultrasound (IVUS) immediately after BMS implantation and at 6 months of follow-up. Both colchicine and placebo were administered orally for 6 months. Serious adverse events were not seen, and colchicine significantly reduced the 6-month angiographic and IVUS restenosis incidence compared to control. Thus, low-dose colchicine was effective in reducing neointimal hyperplasia, thereby lowering ISR rates in a high-risk population.

Unlike sirolimus, colchicine is inexpensive, well tolerated, and widely available, with expanding evidence of cardiovascular benefit. In the Low-Dose Colchicine (LoDoCo2) trial, daily colchicine at 0.5 mg reduced cardiovascular events by 31% among patients with stable CAD. Likewise, the COLCOT trial demonstrated a 23% reduction in major adverse cardiovascular events (MACE) post-myocardial infarction [45,46,49].

These findings prompted an investigation into colchicine’s role as an adjunct to PCI, hypothesizing that attenuating periprocedural and post-implantation inflammation could reduce the risk of restenosis and thrombosis following stent deployment during PCI [49].

In two RCTs, colchicine was given as a single bolus dose before PCI in patients with AMI. In the study by Cole et al., colchicine reduced periprocedural myocardial injury. In the study by Shah et al., colchicine was not able to improve clinical outcomes, although it reduced the inflammatory markers at 24 h after PCI. In all of these trials, colchicine improved clinical outcomes; however, in both studies, the drug was given as a single bolus [50,51].

More recently, two RCTs analyzed the effectiveness and cost-effectiveness of colchicine in the setting of PCI, given orally chronically in one and for 3 months in the other.

In these two trials, colchicine was used as an adjunct to the new DES generation (DES2G) in one and after BMS implantation in the other.

In the Clear Synergy RCTs [33], the addition of colchicine did not improve clinical outcomes in patients with AMI undergoing PCI with the newest DES2G designs compared to DES2G with placebo.

In this trial, investigators randomized 7062 patients at 104 centers in 14 countries. Patients were randomized to PCI with DES2G implantation plus colchicine 0.5 mg × day versus DES2G implantation plus placebo. A primary endpoint occurred in 322 of 3528 patients (9.1%) in the colchicine group and 327 of 3534 patients (9.3%) in the placebo group over a median follow-up period of 3 years (*p* = 0.93). The occurrence of each component of the primary outcome was similar in the two groups. Diarrhea occurred more frequently in patients with colchicine than with placebo (10.2% vs. 6.6%; *p* < 0.001), but the incidence of serious adverse effects or infections did not differ between groups.

#### The ORCA RCTs Trial

The Oral Colchicine After BMS (ORCA) trial, published in 2025 in the Journal of Clinical Medicine, directly compared BMS plus colchicine versus DES2G in 410 patients. Patients in the BMS group received colchicine 1 mg daily for 3 months post-PCI [52,53].

At a mean of 3 years of follow-up [53], rates of MACE (all cause of death, myocardial infarction, cerebrovascular accident, or target lesion revascularization) did not differ between groups (12.7% vs. 15.6% in the colchicine and DES groups, respectively; *p* = 0.39). Ischemic TVR was 9.5% and 9.4% with colchicine plus BMS versus the DES2G groups, respectively. Importantly, implantation with BMS plus colchicine achieved a mean cost reduction of approximately 15% per case (USD 4826 vs. 5708, *p* < 0.001). Adverse effects were minimal and primarily gastrointestinal: diarrhea 5%. The authors concluded that systemic anti-inflammatory therapy could restore BMS viability as a low-cost PCI strategy with clinical efficacy comparable to DES2G Table 3.

Limitations of these findings are described in Table 3. The patient population of ORCA included 75% of acute coronary syndromes (ACS), including MI, and a low Syntax Score (SS).

In the recently reported RCT, Clear Synergy [33], the addition of colchicine did not enhance clinical results. We can only hypothesize about the differences between their outcomes [33,52,53,54]. First, in ORCA, the stent design was BMS in 100% of patients included in the colchicine arm, vs. 0.3% in the Clear Synergy arm.

Intimal hyperplasia during the first year and ISR were the main limitations of the BMS design, and, as previously described, colchicine was shown to significantly reduce them in an RCT [48].

In contrast, endothelial dysfunction and early neo-atherosclerosis observed after DES implantation were seen as the main limitations of the current DES2G [9,10,54]. To our knowledge, the lack of vasomotor response, whether to nitroglycerin or adenosine, observed after DES implantation could not be changed with colchicine.

Secondly, in ORCA, colchicine was administered at 0.5 mg twice daily, which is double the dose recommended in Clear Synergy.

Even though CRP was significantly reduced, it was still at a high therapeutic level of 3 mg/L, which is above the goal of below 2 mg/L. In contrast, in ORCA, the CRP level at 30 days in colchicine was 1.4 mg/L.

## 3. Clinical Considerations

It is well known that DES2G has significantly improved safety and efficacy compared to the first DES or BMS and has become the default strategy during PCI, but this has not translated into a survival benefit for DES2G compared to BMS in the largest RCT ever performed [55]. Also, a recent meta-analysis of RCTs showed a safety benefit of DES2 over BMS only in patients with left main coronary artery (LMCA) or left anterior descending artery (LAD) lesions [56,57]. On the contrary, it did not show a benefit in non-LMCA/LAD lesions, where all-cause death at five years was numerically higher with DES2G [56]. In addition, some low- and middle-income regions, including Latin America, still face economic constraints that limit the widespread use of DES, as reported in a recent regional registry [16]. The forecast of worldwide BMS market expansion in the next 9 years, as shown in Figure 1, aligns with the above findings.

Beyond suppression of post-stenting inflammation, colchicine may favor endothelial healing. Experimental studies indicate that colchicine modulates endothelial progenitor cell mobilization and reduces expression of reactive oxygen species within the vascular wall. Such effects may contribute to reduced neointimal proliferation and improved vessel remodeling after BMS placement.

From a mechanistic standpoint, sirolimus exerts a more direct antiproliferative effect, whereas colchicine primarily modulates inflammation. Their combined or sequential use may theoretically provide synergistic control of restenosis and inflammation. However, clinical evidence evaluating dual therapy remains lacking.

Of interest in the ORCA trial, a comparison was made with newer DES2G designs, and the results were similar to those reported years before with oral sirolimus and prednisone against 1st-DES designs. In Table 1, Table 2 and Table 3 we can see the study designs and long-term outcomes from the 3 RCTs comparing oral anti-inflammatory or immunosuppressive therapy against DES. (ORAR III;CEREA-DES; and ORCA trial, respectively).

In the Graphical Abstract, the overall results and clinical implications are described.

In Table 1 and Table 2, and the Graphical Abstract, we highlight the major limitation of ORAR and CEREA-DES: the use of old-DES designs as comparators.

The negative results of colchicine after implantation of DES2G observed in the Clear-Synergy trial, in contrast to the ORCA trial, demonstrate that ISR due to intimal hyperplasia is the main limitation after BMS implantation, but not after DES [51,52].

Across three decades of Argentinian and European trials exploring oral anti-inflammatory or immunosuppressive adjuncts with BMS:

Each regimen (sirolimus, prednisone, colchicine) produced clinical outcomes comparable to or superior to those of DES at significantly lower cost.

ORAR III demonstrated the strongest long-term mortality advantage; however, as we described above, the high incidence of late stent thrombosis and cardiac death with the first DES designs is no longer observed with DES2G in almost all RCTs and meta-analyses [11,12,53,55,56,57].

However, the ORCA trial, now using DES2G, suggests that oral immunosuppressive therapy plus BMS is cost-saving compared to the DES approach.

Collectively, these imply that BMS + targeted short-term oral therapy remains a viable alternative to DES in highly selected patients. We should take into account that these three trials have had a high number of ACS cases and have included patients with low SS, such as in the ORCA trial; these limitations should be considered when analyzing the trial results and shouldn’t be applied to other clinical scenarios.

The cost of DES varies widely across settings but remains several-fold higher than that of BMS. In many LMICs, patients pay out-of-pocket for coronary interventions, rendering DES implantation financially inaccessible. A pharmacologic BMS optimization strategy—using oral sirolimus or colchicine—offers a pragmatic path toward equitable revascularization.

The unexpected finding of high non-cardiac death incidence after PCI with DES in recent registries and RCTs would be a cause of concern [11,12,13,14,15], and if it is related to DES use, it could be in favor of the concept of systemic pharmacologic BMS optimization.

Oral sirolimus and colchicine, as used in the ORAR and ORCA trials, require no specialized monitoring in patients without contraindications to these drugs and can be easily incorporated into standard post-PCI protocols at both tertiary and district-level hospitals under cardiology supervision.

As we stated before, despite the positive results of prednisone, the exclusion of the diabetic population implies that this drug appears to be, at the present time, less attractive than rapamycin and colchicine.

Finally, in spite of systemic administration of these drugs, major adverse systemic side effects were not seen, maybe due to the short period of administration of the drug.

## 4. Patient Selection

Candidates for BMS plus systemic therapy, BMS-Optimization, may include: Patients with high bleeding risk who necessitate shorter DAPT duration; those expected to undergo non-cardiac surgery soon after PCI; and patients in areas where DES is prohibitively expensive or unavailable.

Individuals with chronic inflammatory or metabolic comorbidities may benefit from anti-inflammatory modulation.

Adoption of oral adjunct therapy aligns with current trends emphasizing personalized and value-based cardiology. The ability to tailor PCI not only to lesion complexity but also to socioeconomic and systemic factors broadens the therapeutic horizon. Importantly, both oral sirolimus and colchicine exemplify drug repurposing—an approach increasingly valuable in pharmacoeconomically constrained health systems.

## 5. Future Directions and Research Needs

Several areas warrant further exploration:

# Large-scale randomized controlled trials to validate clinical equivalence of BMS plus oral sirolimus or colchicine therapy compared to DES2G in diverse populations, taking into account the large proportion of ACS in the three trials (Graphical Abstract).
# Particularly of interest should be the incidence of all-cause of death, cardiac, and non-cardiac, MI, and TVR in both groups.
# Mechanistic investigations of endothelial healing, inflammation resolution kinetics, and local vessel wall drug concentration following systemic administration.
# Vasomotion reactivity after stent deployment at follow-up, with BMS+ adjunctive systemic therapy vs. DES2G, could be an area of special interest for future clinical outcomes of these strategies.
# Health economics modeling to evaluate aggregate cost–benefit under varying healthcare system constraints.
# Evaluation in high-risk subgroups, including diabetics, chronic kidney disease, and multivessel disease.
# Digital adherence monitoring and pharmacovigilance programs to ensure real-world feasibility of oral regimens.

The refinement of such strategies could redefine BMS as a viable platform for cost-effective PCI in a world increasingly mindful of healthcare value.

## 6. Conclusions

The re-emergence of interest in BMS optimization through systemic pharmacologic therapy represents a meaningful step toward reconciling affordability and efficacy in global cardiology practice. Both oral sirolimus and colchicine have demonstrated the ability to mitigate restenosis and adverse cardiovascular events after BMS implantation, in both diabetic and non-diabetic populations.

Although current evidence remains limited to small/moderately sized trials, in contrast to the evidence from many large RCTs with DES, results from the three RCTs described in this revision, which represent the entire RCT data comparing these two therapeutic strategies, provide compelling clinical proof of concept and should be treated as hypothesis-generating. If validated by larger international studies, the systemic pharmacologic stent optimization model could deliver durable, equitable outcomes in coronary revascularization worldwide.

Oral systemic immunosuppression after BMS implantation represents a pharmacologic revival of mechanical stenting—integrating systemic biology into device therapy.

It redefines restenosis prevention as a systemic, rather than strictly local, challenge—bridging affordability, effectiveness, and access in global cardiovascular care.

## Figures and Tables

**Table 1:** ORAR III Trial–Oral Rapamycin + BMS vs. DES. Reference: Rodriguez AE et al [28].

Population: 200 patients with de novo coronary lesions Design: BMS + 14 days oral rapamycin (10 mg loading → thereafter 3 mg/day × 13 days) vs. first generation DES (Taxus, Cypher, Endeavour) Follow up: 5 years End Point: Cost/Effective			
Outcome	BMS + OR (100)	DES (100)	*p*-value
Death	6%	16%	0.02
Death + MI + Stroke	12%	25%	0.01
TLR	10%	17.6%	0.05
Definite/Probable Stent Thrombosis	2%	9%	0.03
Cumulative Cost	Lower	Higher	0.001

ACS 59% including MI; B2C lesions 64.5%; LMCA 5%; MVD 50%; ACS acute coronary syndroms; MI myocardial infarction; LMCA left main coronary artery; MVD multiple vessel disease, OR: oral rapamycin; Interpretation: Oral rapamycin + BMS achieved significantly lower mortality and adverse events at 5 years, with substantial cost savings vs. DES, showing long term superiority. Limitation 1st DES generation.

**Table 2:** CEREA DES Trial–Oral Prednisone + BMS vs. DES. Reference: Ribichini F et al [40].

Population: 375 non diabetic CAD patients Design: 3 arms— BMS + placebo, BMS + prednisone (40 days), DES Primary endpoint: MACE = death, MI, and target vessel revascularization ACS 62.4% including MI Follow up: 4 years				
Outcome	BMS + Prednisone (125)	DES (125)	BMS alone (125l)	*p*-value
Event-free survival (1 yr)	88.0%	88.8%	80.8%	
Event-free survival (4 yrs)	84.1%	80.6%	75.3%	Prednisone vs. BMS = 0.007; DES vs. BMS *p* = 0.03
TVR rate (4 yrs)	13.6%	15.2%	23.2%	
Very-late stent thrombosis and MI	Lower than DES	Higher rate	–	

Interpretation: Prednisone after BMS provided similar or slightly better long term efficacy than DES, with fewer very late events. BMS: bare metal stents; DES: drug eluting stents; MI myocardial infarction; TVR target vessel revascularization; ACS: acute coronary syndroms; CAD: coronary artery disease; MACE: major adverse cardiovascular events. Limitation 1st DES generation.

**Table 3:** ORCA Trial–Oral Colchicine + BMS vs. DES2G. Reference: Rodriguez-Granillo AM et al. [53].

Population: 410 patients with CAD (76% acute coronary syndrome) Design: BMS + oral colchicine (0.5 mg/d twice a day for 3 months) vs. DES2G Follow up: Median 36.8 months (3 years) ACS including MI 75% LMCA 5% MVD 45% SS 21.4% End point cost/effective			
Outcome	BMS + Colchicine(205)	DES2G(205)	*p*-value
Major adverse cardiac & cerebrovascular events (MACE)	12.7%	15.6%	0.78
Death, MI, Stroke	5.4%	9.3%	0.56
Cumulative cost (USD)	4826 ± 2512	5708 ± 3637	<0.001

CAD coronary artery disease; MI myocardial infarction; ACS: acute coronary syndroms; DES2G second generation drug eluting stents; BMS bare metal stent; LMCA left main coronary artery; MVD multiple vessel disease; SS Syntax Score. Interpretation: BMS + colchicine demonstrated comparable 3 year outcomes to DES2G at lower cost, suggesting non inferior efficacy though the trial wasn’t powered for equivalence. Limitations High number of ACS; low SS.

## Data Availability

No new data were created or analyzed in this study. Data sharing is not applicable to this article.
